# Analysis of the Photocatalytic Activity and Adsorption of CuS Nanoparticles Synthesized by Chemical Route for the Degradation of Organic Contaminants

**DOI:** 10.1021/acsomega.5c02366

**Published:** 2025-07-11

**Authors:** Crislaine Beatriz Guedes da Silva, Luciano Lucas Fernandes Lima, Antônio Marcos Urbano de Araujo, Pâmala Samara Vieira, Thércio Henrique de Carvalho Costa, Ramón Raudel Peña Garcia, André Felipe Soares do Monte e Silva, Rômulo Ribeiro Magalhães de Sousa, Amanda Melissa Damião Leite, Maxwell Santana Libório

**Affiliations:** † Department of Mechanical Engineering, 28123 Federal University of Rio Grande do Norte, 59075-000 Natal, RN, Brazil; ‡ Postgraduate Materials Science and Engineering Program, Federal University of Rio Grande do Norte, 59075-000 Natal, RN, Brazil; § School of Science and Technology, Federal University of Rio Grande do Norte, 59075-000 Natal, RN, Brazil; ∥ Graduate Materials Science and Engineering Program, Federal University of Recife, 50670-420 Recife, PE, Brazil; ⊥ Postgraduate Materials Science and Engineering Program, Federal University of Piauí, 64049-550 Teresina, PI, Brazil

## Abstract

The treatment of industrial and domestic wastewater is an urgent environmental need. In this context, the photocatalytic activity of semiconductors offers a promising route for degrading organic contaminants. CuS nanoparticles were chemically synthesized using thiourea and copper sulfate in varying concentrations to investigate how precursor ratios affect the chemical composition, structural and morphological features, and optical-electronic properties. The photocatalytic degradation of methylene blue under low-power visible light (10 W), without H_2_O_2_ and using a low catalyst dose, showed promising results. Samples with lower sulfate content reached ∼78% degradation, while those with 0.20 M thiourea and 0.15–0.20 M sulfate achieved up to 99%. Mesoporous and macroporous structures (3.85–50 nm) promoted adsorption without hindering photocatalytic efficiency, indicating that, in certain samples, the combined morphological and electronic features enhanced dye removal.

## Graphical abstract

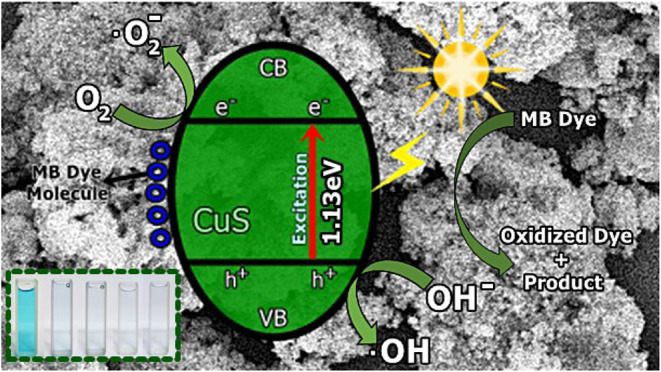

## Introduction

Due to the environmental risks posed by the release of organic and harmful pollutants into the water by accelerated industrial development, mainly in the pharmaceutical, textile, and domestic waste sectors, there is a need for safe, simple, and inexpensive technological improvements to eliminate contaminants, reusing liquid waste in other industrial processes, and consequently purifying water.^1,2^

Approximately 20% of the dyes produced by industry daily are discharged into water as effluents. If not properly treated, this waste can pose severe risks to human and animal health.^1,3^ Therefore, due to the serious environmental problem and current energy challenges, the chemical degradation of these contaminants using semiconductor particles irradiated with visible light presents itself as a nontoxic, sustainable, and promising alternative.^4,5^ However, photocatalytic processes can become economically unviable when they depend on artificial radiation sources or when the penetration of radiation into the reaction medium is limited.^6^

Copper sulfide semiconductor nanoparticles (CuS NPs) have garnered significant attention in recent years due to their exceptional optical, electrical, and chemical properties, making them highly versatile for various technological applications.^7^ These include photocatalysis,^2,8^ photovoltaics,^9^ sensing devices,^10^ and even biomedical treatments such as cancer therapy.^11^ The usability of this material stems from its tunable properties, which are intrinsically linked to its stoichiometry, crystallinity, size, and morphology.^7^ A wide bandgap energy range (1.0–2.5 eV) (without taking into account the effects of plasmon resonance and quantum confinement due to the dimensions of the NPs^12^), resulting from differences in optical-electrical behavior, provides different functionalities for this semiconductor.^13,14^ In addition, the ease of synthesis using economical methods such as precipitation in a chemical bath allows for scalability and commercial integration.^15^

Jan et al., 2025, used a chemical coprecipitation route to produce CuS NPs from CuCl_2_ and Na_2_S as precursors for the Cu and S ions. With a bandgap equal to 2.1 eV, the semiconductor was used to remove 2-chlorophenol in the visible region. The results showed a degradation of approximately 83.2% of the residue.^16^ Iván Nancucheo et al., 2022, also produced CuS nanostructures by biological reduction of sulfate (CuSO_4_) with a direct bandgap between 2.12 and 2.14 eV. Three mg of CuS-NPs were added to 10 mL of methylene blue (MB) solution with a concentration of 5 × 10^–5^ M (6.0 × 10^3^ g/mol) and aided by the reducing agent NaBH_4_ to optimize photocatalysis with a 600 W halogen lamp. The authors reported a maximum degradation of 77.9% in 25 min of analysis, with no subsequent variations. Several other studies have reported contaminant degradation tests with high-power lamps, using reducing reagents or electron–hole recombination (e^–^/h^+^) inhibitors such as H_2_O_2_ or higher g/mol mass ratios.^100–19^ Furthermore, there is a lack of discussion regarding the adsorption of contaminant molecules by the NPs during the degradation process.

Therefore, this study aims to analyze the composition, crystal structure, optical-electrical properties, dimensions, adsorption, and photocatalysis of the NPs as a function of the concentrations of the Cu (CuSO_4_·5H_2_O) and S (NH_2_CSNH_2_) ion precursors produced by a simple chemical route. For the MB solution degradation analysis, a simplified system was adopted, consisting of a low-power halogen lamp, no use of H_2_O_2_, and a low concentration of NPs in relation to the contaminant to analyze the adsorption and photodegradation effects under low reactive stimulus conditions. This system aims to simulate an environment that presents unfavorable conditions for the physical and chemical phenomena necessary for contaminant degradation and to show the pure efficiency of CuS NPs.

## Materials and Methods

The CuS nanoparticles were produced using a chemical bath synthesis route, as shown in the diagram in Figure 1. The analytical-grade chemical reagents used in this synthesis method were thiourea (TU) (NH_2_CSNH_2_) and copper sulfate pentahydrate (CuSO_4_·5H_2_O) as precursors for the elements copper and sulfur, and ammonium hydroxide (NH_4_OH) to control the pH of the copper sulfate solution. TU and copper sulfate were dissolved separately in 100 mL of distilled water, both undergoing the following possible reactions, such as ionic dissolution (1) and alkaline hydrolysis (2–3). Ammonium hydroxide was then slowly dripped into the copper sulfate solution (4), leaving it at pH 9 (blue solution). The TU solution was slowly added to the copper sulfate solution to form a single solution with a volume of 200 mL (green solution). The solution was then placed in a water bath at 75 °C with magnetic stirring for 60 min, during which the formation of CuS occurred according to 5.^15,20^ The NPs samples were synthesized from solutions of thiourea and copper sulfate at different concentrations. Sample names and characteristics of the syntheses are detailed in Table 1. After the NPs production, the particles are separated from the liquid residue by filtering with 80 g/m^2^ paper and pores with an average diameter of 8 μm. Finally, the particles are dried at 60 °C and weighed to obtain the production yield in grams per synthesis.

1 $${CuSO}_{4}\cdot 5H_{2}O\left(aq\right)\to {Cu}^{2+}\left(aq\right)+{SO}_{4}^{2-}\left(aq\right)+5H_{2}O$$

2 $$NH_{2}\mathrm{CSN}H_{2}+{\mathrm{OH}}^{-}\to {\mathrm{CH}}_{2}N_{2}+H_{2}O+H_{2}S↑$$

3 $$H_{2}S↔S^{2-}+2H^{+}$$

4 $${\mathrm{Cu}}^{2+}+{4\mathrm{NH}}_{3}↔{[\mathrm{Cu}\left(NH_{3}{)}_{4}\right]}^{2+}$$

5 $${\mathrm{Cu}}^{2+}\left(\mathrm{aq}\right)+S^{2-}\left(\mathrm{aq}\right)\to \mathrm{CuS}\left(s\right)↓$$

**Figure 1:**
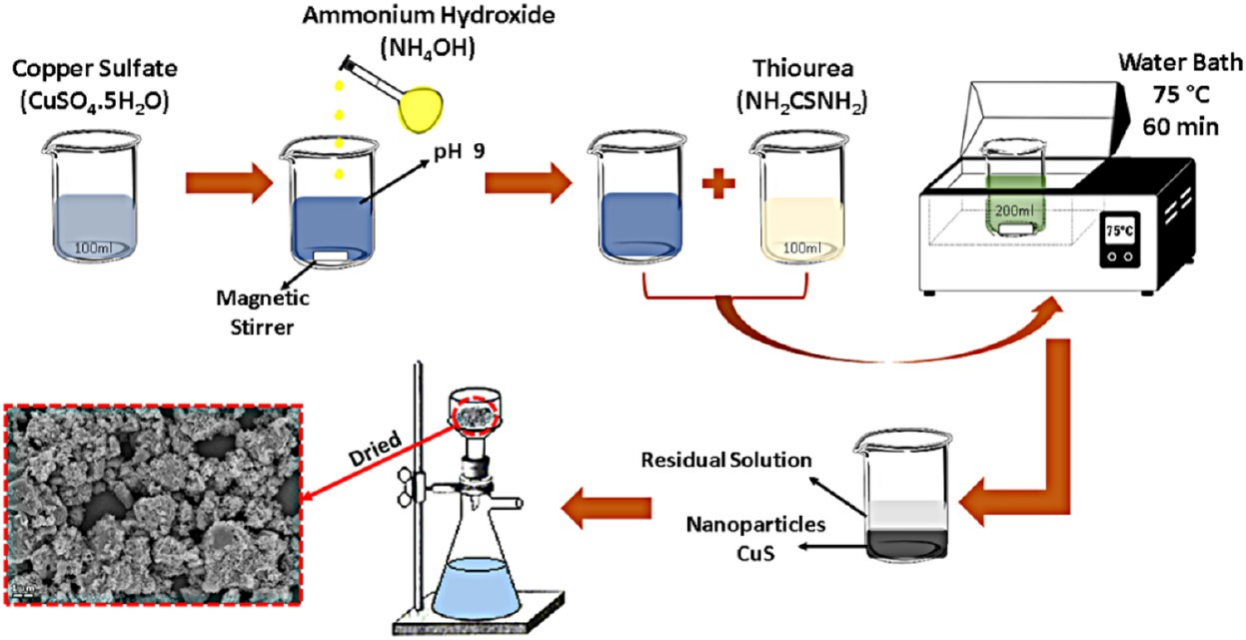
Schematic diagram of the nanoparticle synthesis process.

**Table 1:** Names of the Samples and the Respective Concentrations of the Thiourea and Copper Sulfate Solutions (100 mL)

samples	thiourea concentration (M)	copper sulfate concentration (M)	thiourea mass (g)	copper sulfate mass (g)
T10C10	0.10	0.10	0.76	2.49
T10C15		0.15		3.94
T10C20		0.20		4.98
T10C40		0.40		9.96
T20C10	0.20	0.10	1.52	2.49
T20C15		0.15		3.94
T20C20		0.20		4.98
T20C40		0.40		9.96
T30C10	0.30	0.10	2.28	2.49
T30C15		0.15		3.94
T30C20		0.20		4.98
T30C40		0.40		9.96

The crystallinity of particles was characterized with a BRUKER X-ray diffractometer (DRX) (Model D2 PHASER) with copper Kα radiation (λ = 0.1541 nm), scan step of 0.01°, 20–70°. TOPAZ 4.2 software from Bruker was used to refine the XRD data.

Raman spectroscopy was carried out using a SENTERRA spectrometer (Bruker, Germany) with a He–Ne laser with 15 mW, a wavelength of 532 nm, a 5% power filter, a 10 s acquisition time, and an accumulation of 5 measurements with a spectral range of 200–600 cm^–1^. Ultraviolet–visible (UV–vis) diffuse reflectance spectroscopy (DRS) was performed using a Shimadzu UV–vis spectrometer UV-2700 to examine the optical properties. The energy of the band gap (*E*_g_) was determined using a Tauc graph, using 1.^21^

6 $${\left(\alpha h\nu \right)}^{n}=A\left(h\nu -E_{g}\right)$$

where α, *h*, ν, and *A* are the absorption coefficient, Planck’s constant, frequency, and a constant, respectively.

The following relation calculates the refractive index of the particles:^22^

7 $$\eta =\left(\frac{1+R}{1-R}\right)-\sqrt{\frac{4R}{{\left(1-R\right)}^{2}}-k^{2}}$$

Where η is the refractive index, *k* = αλ/4π is the extinction coefficient, and *R* is the reflectance. The photoluminescence (PL) spectra were excited at room temperature by the 325 nm line of a He–Cd laser using a Jobin Yvon Fluorolog-3 170 spectrofluorometer.^21^ The surface areas were obtained using the Brunauer, Emmet, and Teller (BET) method considering the IUPAC recommendations,^23^ and the pore diameter distribution were calculated by the t-plot method using the Harkins-Jura-de Boer t-equation.^24^

The morphology and elemental composition were analyzed using ZEISS AURIGA 40 field emission scanning electron microscopy (SEM-FEG) coupled with energy dispersive X-ray spectroscopy (EDS). For this analysis, the NPs were dispersed in acetone, ultrasonicated, and dripped onto a silicon plate for evaporation in an oven. Images were also obtained using a Tescan Mira 4 scanning electron microscope in STEM mode using a Bright Field (BF) detector with an acceleration of 30 kV.

The photocatalytic experiment was conducted in a homemade reactor (Figure 2), and 100 mL of a 5 × 10^–5^ M MB solution was used in the photocatalysis test. 20 mg of catalyst (NPs) were added to the MB solution (4.0 × 10^3^ g/mol), which was then subjected to ultrasonic agitation for 1 min to ensure complete dispersion of the particles in the solution and facilitate the adsorption/desorption equilibrium of MB on the catalyst surface. The solution was then kept in the dark box for 30 min with gentle mechanical stirring to collect 5 mL of MB solution and measure the optical absorption (Dark measurement).^25^ The solution was then exposed to the controlled illumination of a 10 W halogen lamp with predominant emission at λ = 440 nm and a distance of 20 cm between the light source and the solution’s surface. Aliquots of 5 mL of the MB solution were removed from the container and centrifuged for 5 min at 5000 rpm to determine the absorbance (*A*) of the solution by UV–vis spectrophotometry at times of 30, 60, 90, 120, 150, 180, and 210 min. The concentration of MB in the solution was monitored by changing the intensity of the absorption peak at a wavelength of 664 nm, using a Genesys 10UV Scanning spectrophotometer (Thermo Scientific) where *A*/*A*_0_ = *C*/*C*_0_. A representation of the photocatalysis system is shown in Figure 2.

**Figure 2:**
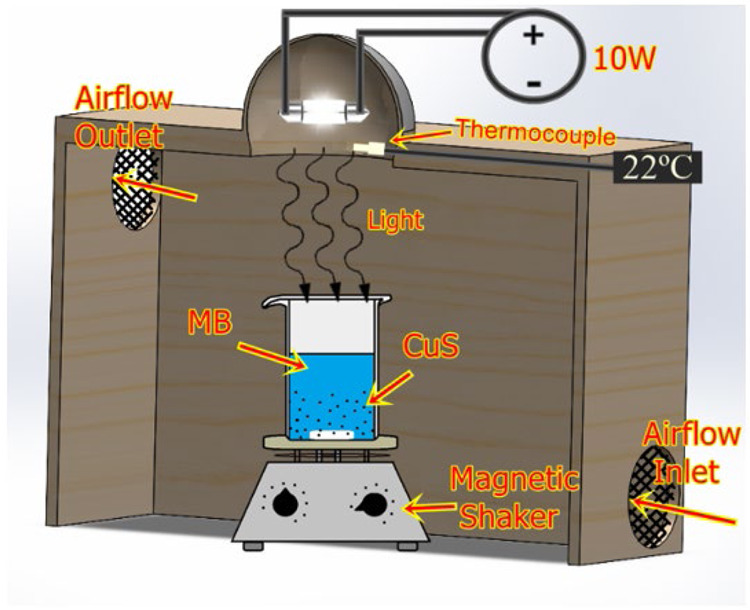
Diagram of the photodegradation oven.

## Results and Discussion

Figure 3 shows the powder mass produced in each synthesis defined by combining different concentrations of the copper ion (copper sulfate) and sulfur (thiourea) precursor reagents to form the CuS phase. It can be seen that the variation in the concentrations of the reagents significantly influences the quantity of particles produced. Lower concentrations of both precursors (e.g., T10C10 and T20C10) yield a small mass. On the other hand, high concentrations contribute to forming more particles during synthesis (T10C40, T20C40). The increase in copper and sulfur ions available for nucleation and formation of the solid phases of the semiconductors can explain this. These data also show that increasing the concentration of copper sulfate influences the nucleation of solid phases more strongly than thiourea, indicating a possible saturation of sulfur ions and consequent formation of copper-rich phases.

**Figure 3:**
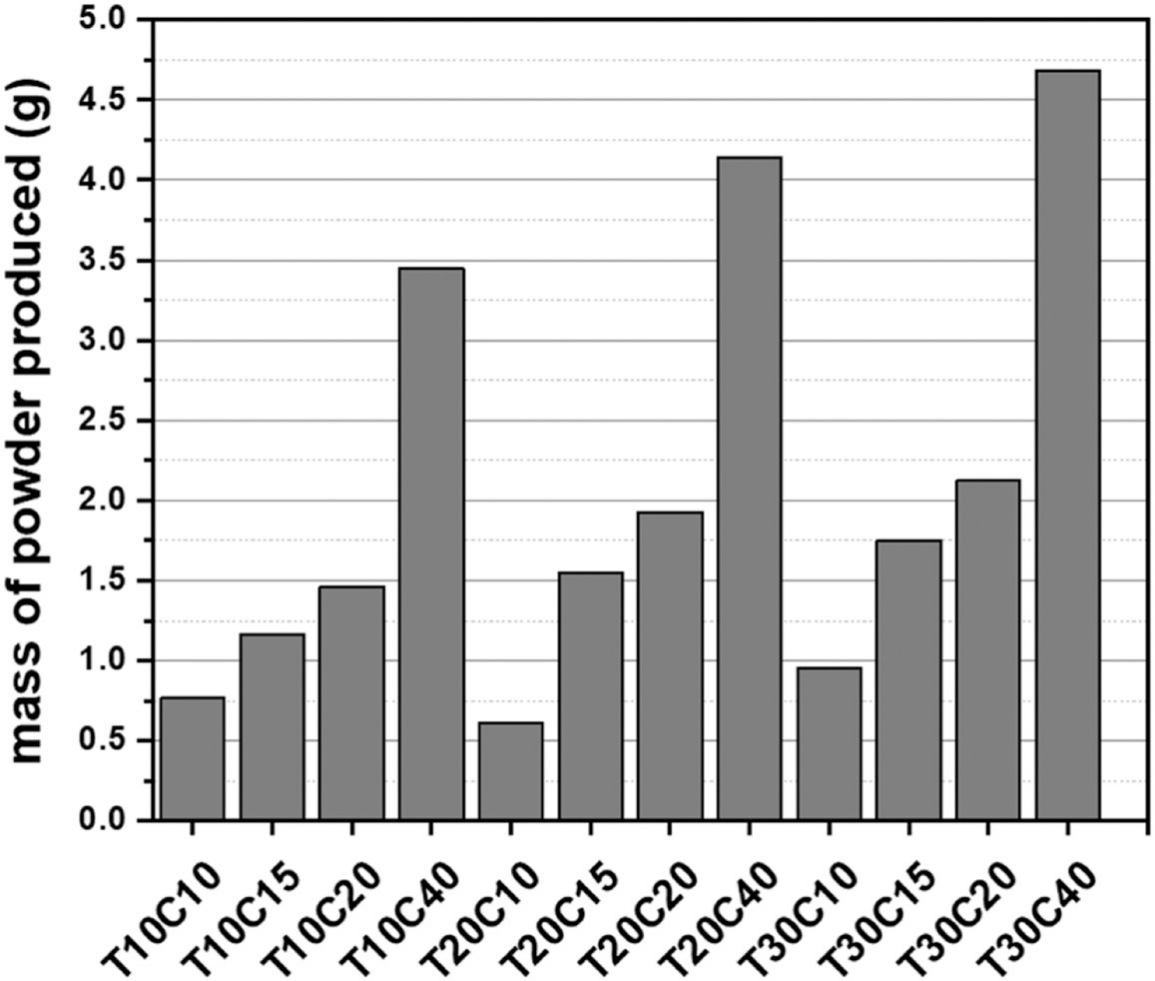
Mass of particles produced in each chemical synthesis.

Figure 4 shows the X-ray diffractograms of the samples synthesized by the chemical bath. The samples synthesized with 0.1, 0.15, and 0.2 M copper sulfate solutions showed diffraction peaks at positions 29.54° (102), 32.07° (103), and 48.37° (107) referring to the reflections of hexagonal CuS phases (ICSD 32106) with low crystallinity, which points to the formation of nanostructured particles.^25,26^ On the other hand, the peaks located in other positions with a higher degree of crystallinity are phases of a hydroxy sulfate formed by the oxidation of copper sulfate, monoclinic Cu_4_(OH)_6_SO_4_, known as Brochantite (ICSD 59288).^27^

**Figure 4:**
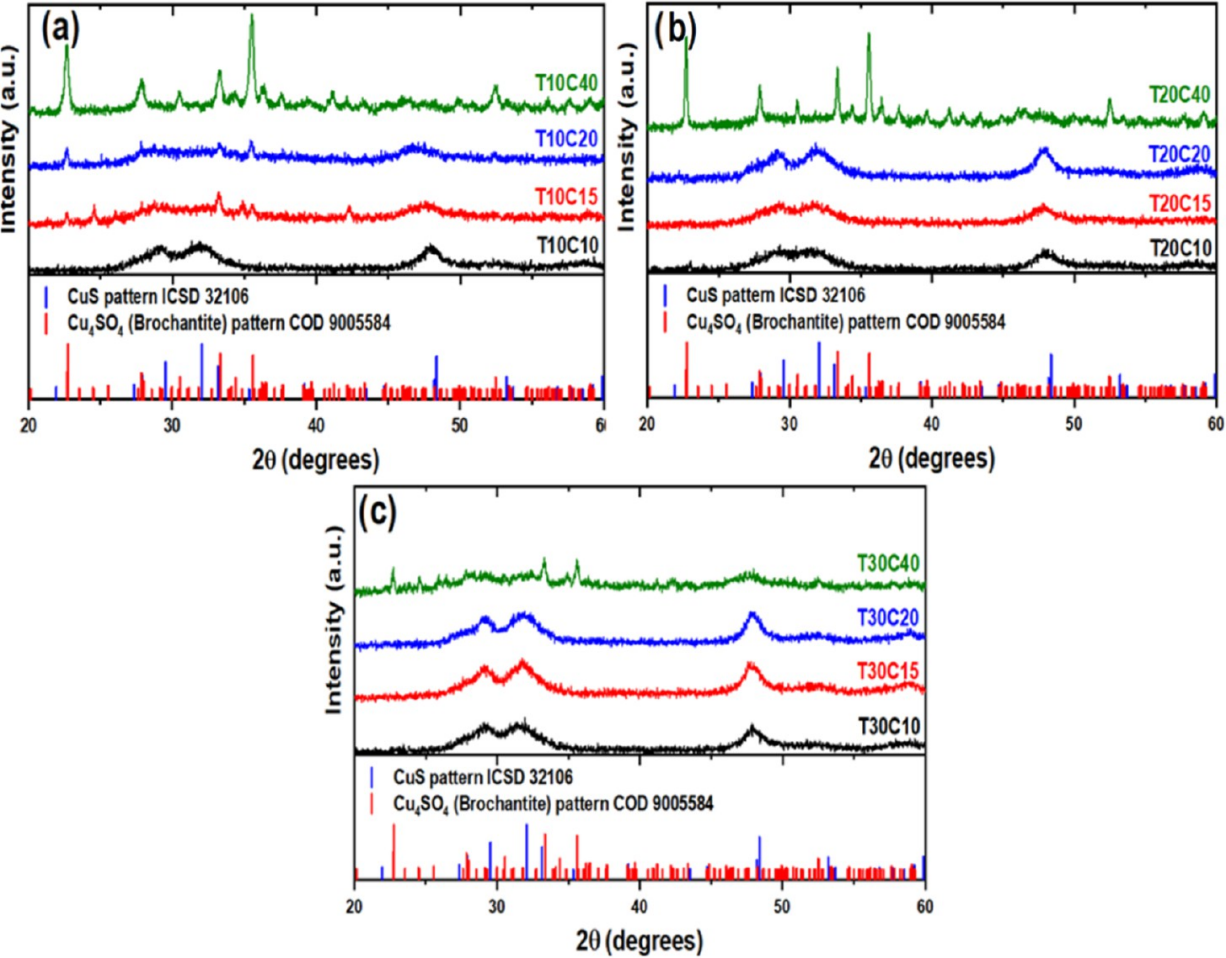
Diffractograms of all the synthesized samples: samples with (a) 0.1 M, (b) 0.2 M, and (c) 0.3 M TU.

Figure 4a shows that samples T10C15 and T10C20 had less intense CuS phases and, in addition, secondary phases of brochantite. This oxidized phase is predominant in sample T10C40 and could, therefore, be a detrimental factor for the material’s performance as a catalyst during methylene blue photodegradation tests (example of textile waste). Increasing the concentration of thiourea to 0.2 M significantly reduced the formation of secondary phases in samples with a copper sulfate concentration of up to 0.2 M (T20C20) but contributed to the formation of even more crystalline brochantite in T20C40. Figure 4c shows the NPs synthesized with 0.3 M thiourea. In these cases, the reflections referring to the CuS phase are even more defined than in the previous cases, and the diffractogram of sample T30C40, unlike the other NPs produced with 0.4 M CuSO_4_·5H_2_O, results in the formation of CuS and phases from the growth of brochantite.

The formation of brochantite in syntheses with a high concentration of copper sulfate occurs due to the excess of unstable Cu ions in the solution, which undergo oxidation; this process produces cuprite (Cu_2_O), which reacts with the S^2–^ present in the aqueous solution.^28^ According to Zittlau et al., chemical syntheses with a higher concentration of CuSO_4_·5H_2_O result in the precipitation of brochantite.^29^

The XRD data were refined using the Rietveld method. Figure 5 shows the results for samples T10C10 (Covellite only), T20C40 (Brochantite only), and T30C40 (Covellite + Brochantite) to visually exemplify the quality of the refinement procedure.

**Figure 5:**
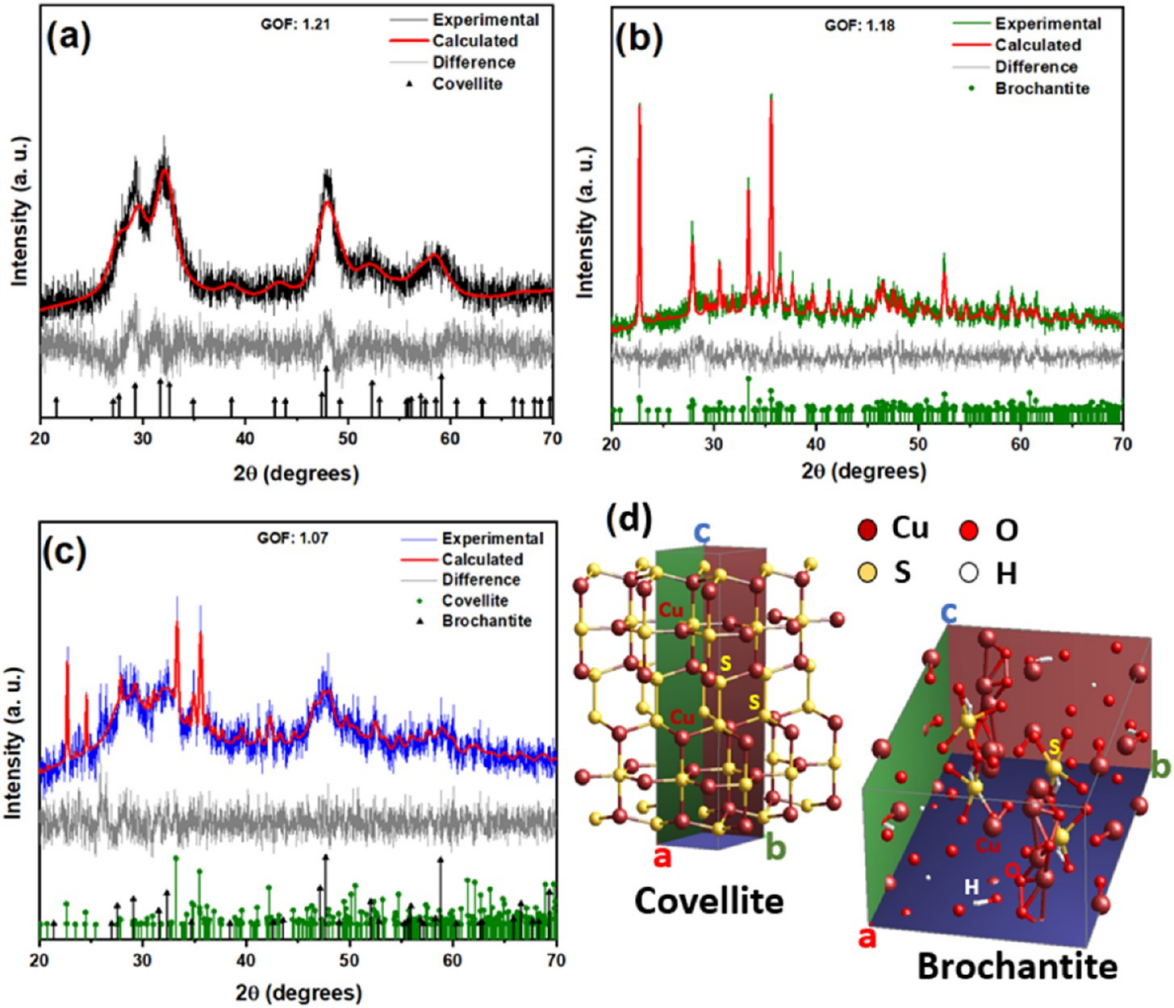
Results of the Rietveld refinement of the samples (a) T10C10, (b) T20C20, (c) T30C40, and (d) representation of the Covellite and Brochantite cells.

The XRD data were refined using the Rietveld method. To visually illustrate the quality of the refinement procedure, Figure 5 shows the results for samples T10C10 (Covellite only), T20C40 (Brochantite only), and T30C40 (Covellite + Brochantite). In all cases, the refinement’s quality of fit (GOF) showed values close to 1.00, confirming the correspondence between the experimental and calculated data.^30^ This is also confirmed by the constant behavior of the residue produced between these data (gray curve). Figure 5d shows an illustration of the Covellite (Hexagonal) cells composed of alternating CuS_3_–CuS_3_–CuS_3_ and S–S bonds along the C axis^31^ and Brochantite (Monoclinic) generated by the TOPAS 4.2 refinement program composed of Cu, S, O, and H atoms.

Table 2 shows the data obtained from the refinement, such as the structural parameters, cell volume, crystallite size, and phase percentage. The structural parameters of the refined phases show that the Covellite and Brochantite phases have undergone structural changes, resulting in different cell volumes. This is due to the synthesis conditions, which favor the formation of structural defects such as vacancies and substitutions. During the formation process of CuS NPs, sites occupied by sulfur (S) can be replaced by oxygen (O) atoms, making the network parameters larger due to the larger size of the oxygen atom.^15,32^ This anionic substitution can result in a change in the band gap and, consequently, in the photocatalytic efficiency of the material.

**Table 2:**
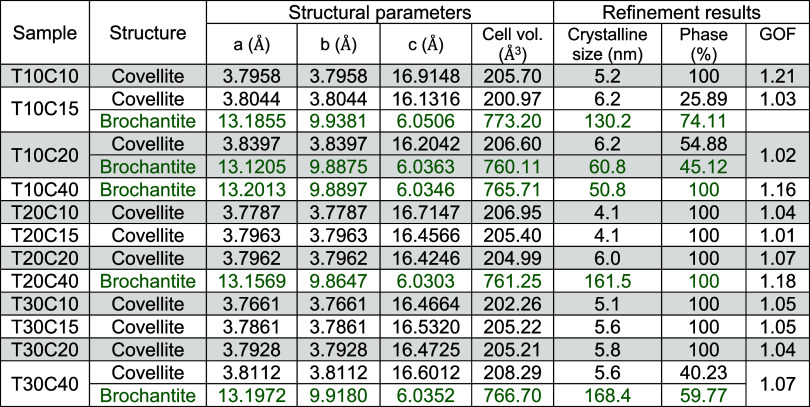
Rietveld Refinement Results: Network Parameters, Crystallite Size, and Phase Percentage

The crystallite sizes obtained by refinement confirm that the Covellite produced is nanometric, as stated by Das et al., 2024, and other publications.^33,25^ This justifies the broad CuS peaks observed in the diffractograms of sample T10C10 (Figure 5a).^34^ Particles of 4.1 to 6.2 nm are obtained, resulting in a very high surface area/volume ratio, which, according to Sabri Ouni et al. and other authors, favors the adsorption effect of the compound to be degraded during tests and chemical reactions due to the exposure of more catalyst sites.^34–36^ Nanostructured particles with these dimensions can also be useful for applications in Plasmonics, as reported by Chen et al., 2021.^37^ The presence of Brochantite due to the excess of Cu^2+^ copper ions in the synthesis is quantified by analyzing the diffractograms.^38^ The crystallite size of Brochantite varied between 50.8 and 168.4 nm, justifying larger lattice parameters and a possible clearer visualization of this phase through scanning microscopy. This crystalline phase, recognizable by its green color, is common on the surface of oxidized copper statues such as the Statue of Liberty. It is reported in studies on magnetic properties, but does not effectively contribute to photocatalytic activity like Covellite.^39^

The Raman spectra of the samples synthesized by the chemical route are shown in Figure 6. In the Raman shift range between 200 and 600 cm^–1^, two characteristic zones of the vibration modes corresponding to the bonds present in Covellite and Brochantite stand out. An accentuated vibration band around 470 cm^–1^, a low-frequency region characteristic of metallic bonds, is attributed to the A_1g_ mode, which corresponds to the stretching and coupling of the S–S bonds of the S_2_ ions, which can be seen in the central region of the structural representation of Covellite shown in Figure 5d.^22^ The Raman spectra showed that increasing thiourea concentration in the particle synthesis process contributed to the formation of more detectable S–S bonds. This means that the increase in thiourea promoted greater purity of CuS since these bonds are rarely present in Brochantite structures, as illustrated in Figure 5d. This is also confirmed by the fact that Brochantite phases do not appear in the diffractograms of the samples synthesized with 0.10, 0.15, and 0.20 M in Figure 4b and c.

**Figure 6:**
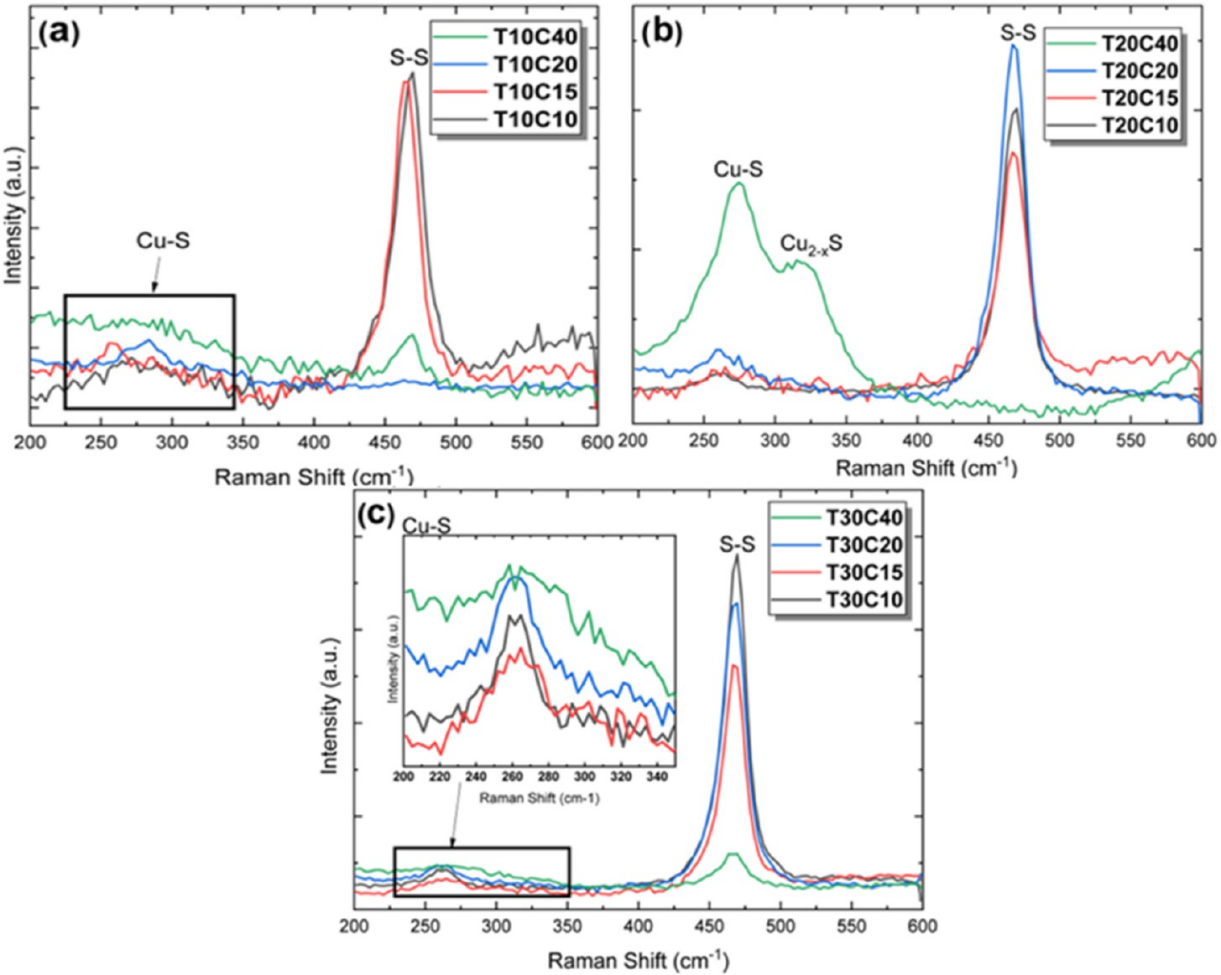
Raman spectra of samples with different compositions. Samples with (a) 0.1 M, (b) 0.2 M, and (c) 0.3 M TU.

The Raman spectrum revealed another peak around 266 cm^–1^ due to the transverse optical mode (TO) of the CuS phase. The position and intensity of these Raman bands that appear in the low-frequency region depend on the intensity of the bond and the size of the nanoparticles.^25,40,41^ In addition, an extra peak in sample T20C40 at 322 cm^–1^ characterized in Figure 6b can be attributed to the Cu_2–*x*_S phonon mode (0.6 ≤ *x* ≤ 1), showing the presence of Brochantite, which is a copper-rich phase. Samples T10C40 and T30C40 show a broad band in this region, which may represent a convolution of more than one band in which Cu_2‑x_S participates. Despite this, the laser did not sensitize other sulfate and hydroxyl (OH) vibration modes in Brochantite because they are weaker or less resonant.^42^

The sharp S–S peaks show that the lattice atoms are aligned in a periodic matrix and that the reduction in the width at half height (FWHM) of the peak represents the growth of the nanostructure and the reduction of the amorphous phase.^43^ As a result, it can be seen that the FWHM values (Table 3), on average, decrease with the increase in thiourea. This result aligns with the appearance of the diffractograms shown in Figure 4, where the peaks tend to become more defined with the increase in thiourea in the reagent solution.

**Table 3:** Calculation of the FWHM of the Vibration Bands of the S–S Bonds

samples	FWHM
T10C10	25.38648
T10C15	22.45229
T10C20	
T10C40	19.64415
T20C10	22.51189
T20C15	21.2886
T20C20	23.09431
T20C40	
T30C10	21.23358
T30C15	20.87826
T30C20	22.06583
T30C40	27.10823

Figure 7 shows the SEM and EDS mapping of samples T10C40 and T20C20, which contain different structures due to the presence of brochantite in the sample synthesized with 0.40 M CuSO_4_·5H_2_O. Some needle-shaped crystals are observed in the brochantite agglomerates (Figure 7a), while more rounded shapes are seen in the samples with a predominance of CuS (Figure 7b).^44^ The mapping showed where the elements that make up the structures (Cu, S, and O) are concentrated. The mapping and EDS results show that the Covellite structure has a higher sulfur content than Brochantite, which has a higher relative percentage of Cu and O due to its Cu_4_(OH)_6_SO_4_ stoichiometry being rich in these elements. The mapping in Figure 7b shows a percentage of 20.05% oxygen in the Covellite phase, which is due to the presence of Brochantite in low quantities and the subsequent oxidation process.

**Figure 7:**
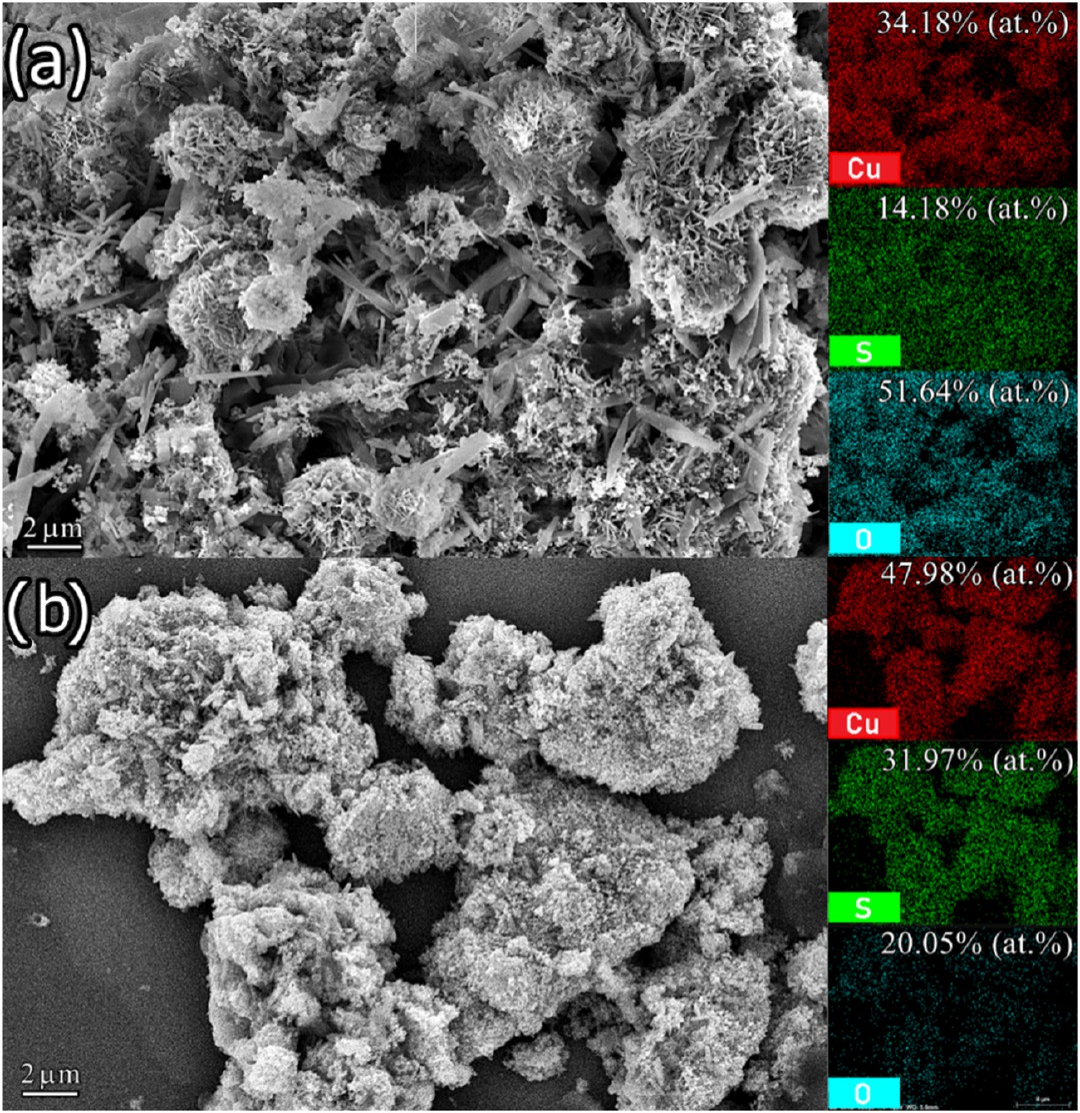
SEM images of the samples: (a) Brochantite (T10C40) and (b) CuS (T20C20), accompanied by the elemental mappings carried out by EDS, with the respective atomic weight percentages of the elements identified.

Figure 8 shows an enlarged micrograph of sample T20C20. Figure 8a shows the presence of coexisting Covellite and Brochantite. This justifies the considerable percentage of oxygen in sample T20C20 in Figure 7b. The elemental mapping in Figure 8a shows that the oxygen is concentrated in the Brochantite crystals. Figure 8b shows the micrograph of particles from sample T20C20 agitated in ultrasound and dispersed on a silicon slide. The image of a small grain detached from the mass of the synthesized material illustrates the ability of the CuS nanoparticles to agglomerate due to the lack of control over the particle growth process.^15^

**Figure 8:**
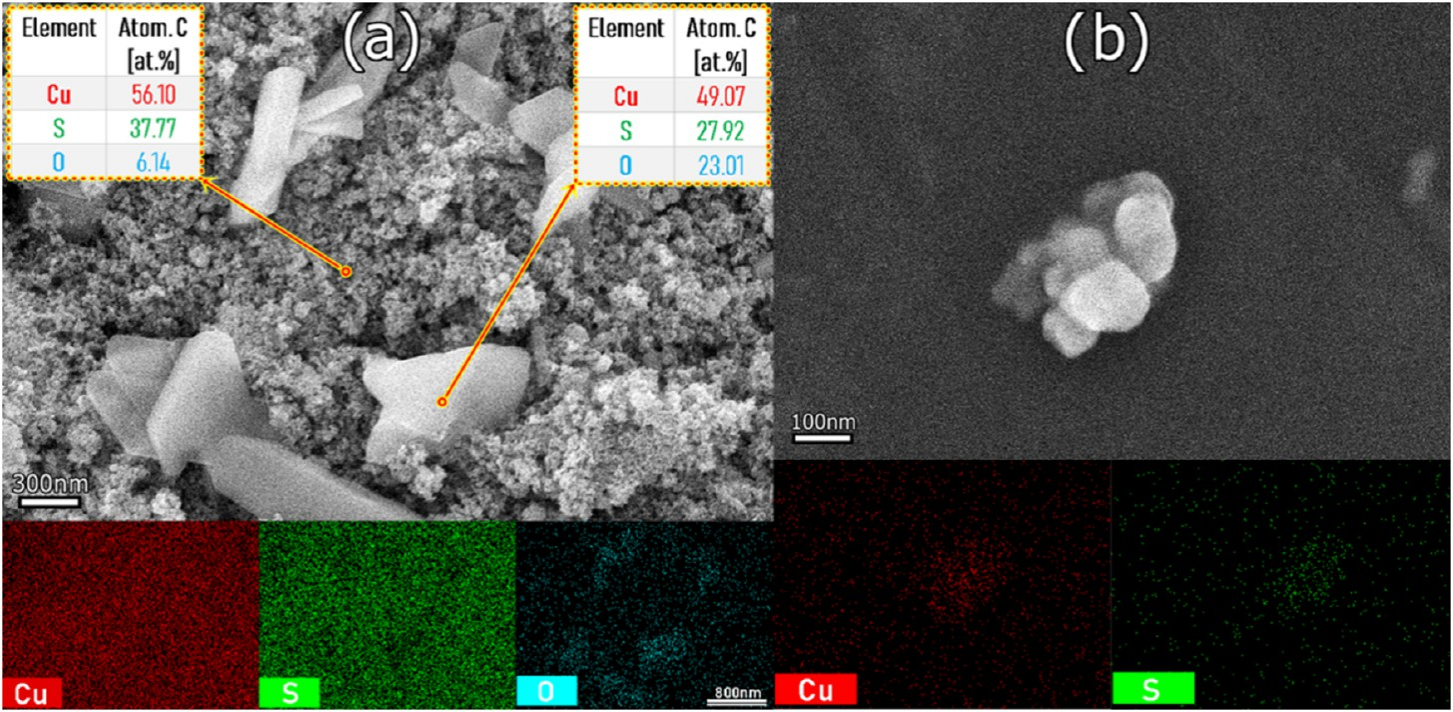
SEM, EDS, and elemental mapping of sample T20C20. (a) Covellite and Brochantite morphology, (b) Small cluster of CuS nanoparticles.

STEM was also used to analyze the morphology and crystallographic characteristics of a cluster of particles from sample T20C20 (Figure 9). The central and darker region, where particle agglomeration is greatest, is too electron-dense for the electron acceleration achieved during analysis (30 kV) in TE-BF mode.^45^ On the other hand, in Zone 1 (Z1) and the enlarged highlight containing zones 2 and 3 (Z2 and Z3), specific crystallographic structures can be observed. Z1 and Z3 (rod) consist of crystalline structures with preferential orientation along a specific direction. On the other hand, Z2 presents a nanocrystalline agglomerate (Figure 9b). This zone represents the broadest form of particle agglomeration, since the diffractogram of this sample shows low crystallinity associated with lower CuS order.

**Figure 9:**
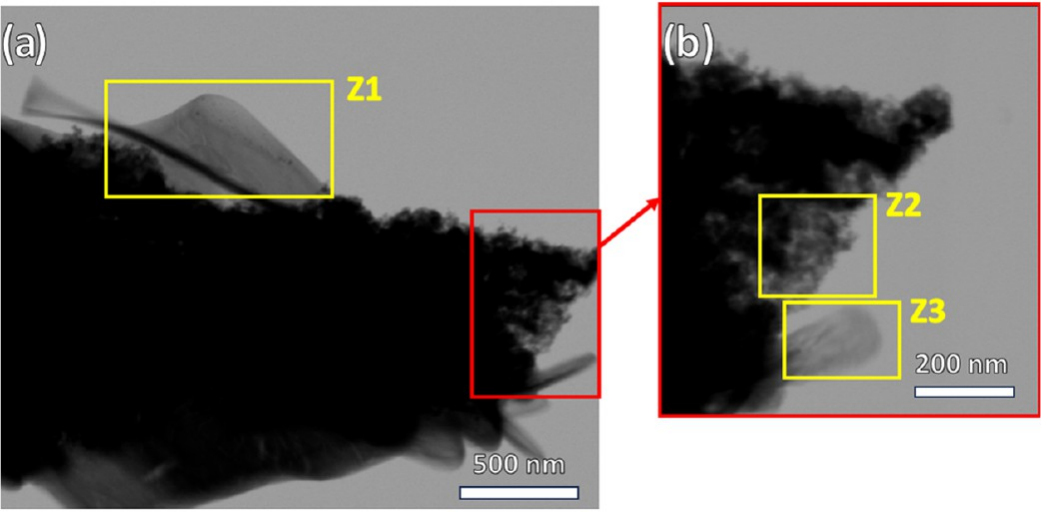
STEM images of a cluster from sample T20C20: (a) larger region, (b) magnification of peripheral region.

Figure 10 shows the results of the Brunauer–Emmett–Teller (BET) principles for the synthesized samples, except those predominantly containing Brochantite. Figure 10a shows that the surface area values of the samples varied considerably depending on the synthesis parameters. For example, samples T20C10 and T30C15 have surface areas of 4.99 and 39.6 m^2^/g, respectively. This variation can be explained by the behavior of the N_2_ adsorption–desorption isotherms of the CuS samples and by the pore size distribution obtained by the Barrett–Joyner–Halenda (BJH) method, which are significant parameters in the adsorption properties and catalytic active sites of the materials.^46^ Figure 9b shows the type IV isotherms of samples T20C10, T30C15, T20C15, and T10C20 (IUPAC classification).^23^ In this type of interaction between particles and N_2_ gas, capillary condensation occurs, accompanied by hysteresis. The shapes of the hysteresis loops observed suggest H2-type loops, characteristic of more complex pore structures.

**Figure 10:**
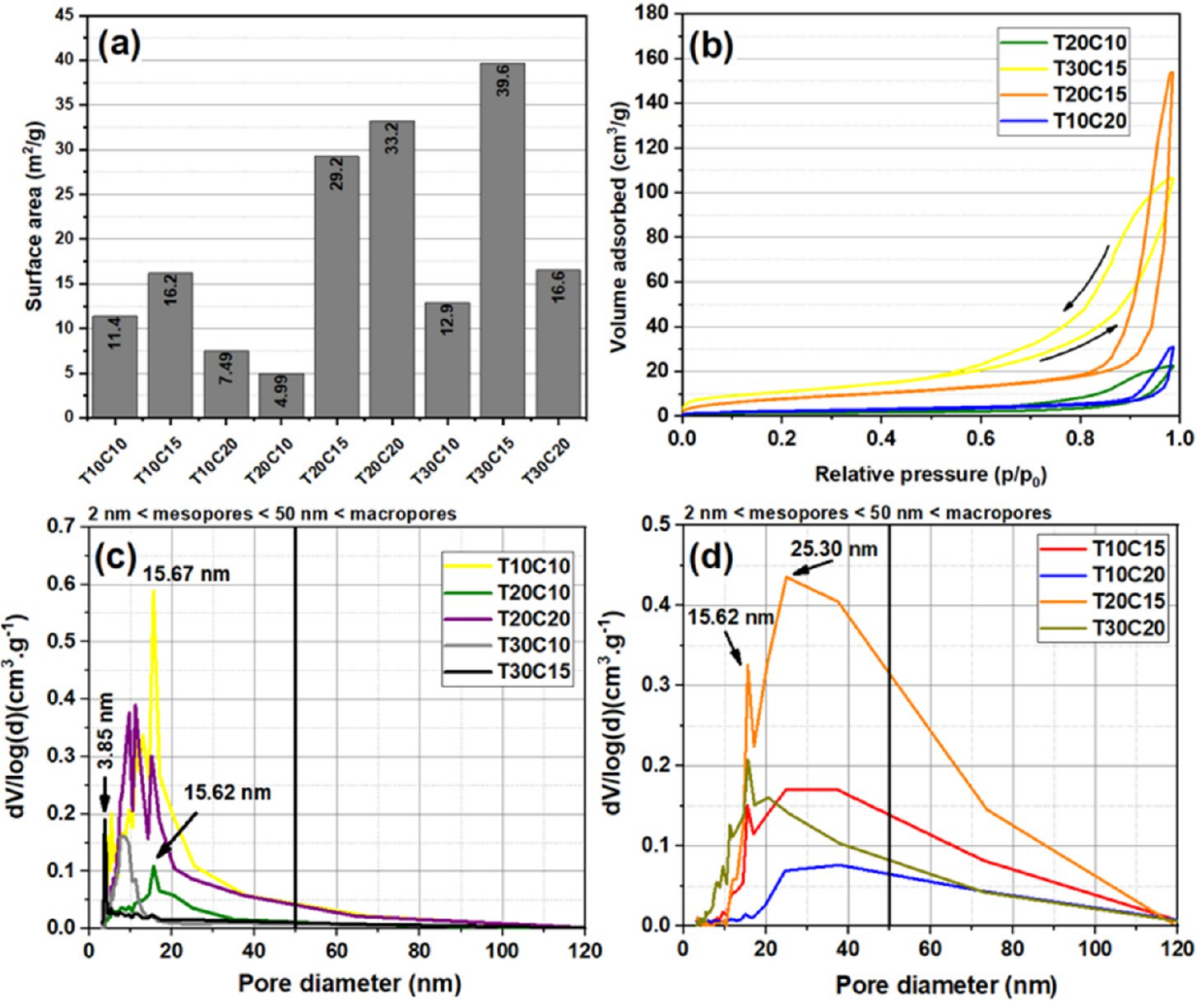
BET and BJH results: (a) specific surface area, (b) N2 adsorption–desorption isotherms, and pore size distribution curves for the least (c) and most dispersed (d) pores.

The very steep desorption branch of sample T20C15, which is characteristic of H2 curves, can be attributed to pore blockage or cavitation-induced evaporation. The presence of type IV hysteresis at relative pressure (*p*/*p*_0_) between 0.6 and 1.0 indicates the presence of mesopores that were likely formed by the agglomeration of small nanoparticles, as explained by Pal et al., 2015.^25^

Figure 10c and d show the distributions of the average pore size. It can be observed that in all the analyzed samples, the formation of mesopores (diameters ranging from 2 to 50 nm) predominates. The samples shown in Figure 9c stand out, with distributions concentrated at values less than 20 nm in diameter. On the other hand, the samples in Figure 9d show a more dispersed distribution, with some macropores (>50 nm) reaching values slightly greater than 100 nm. According to Yu et al., 2010, this dispersion may result from the formation of larger pores produced by interaggregated particles.^47^ These surface area and mesopore dimensions favor the adsorption of large molecules, such as dyes, allowing them to reach the active sites along with light.

The diffuse reflectance technique was employed to determine the band gap values of the produced samples. Figure 11 shows, in general, that the diffuse reflectance decreases with increasing wavelength. However, most samples have a reflectance peak between 500 and 600 nm.^48^ The peak shifts from the dark green color to the lighter region (close to yellow) as the concentration of copper sulfate in the solution increases.^49^ This is visually noticeable since Covellite has a dark green hue and Brochantite has a light green hue.^50^

**Figure 11:**
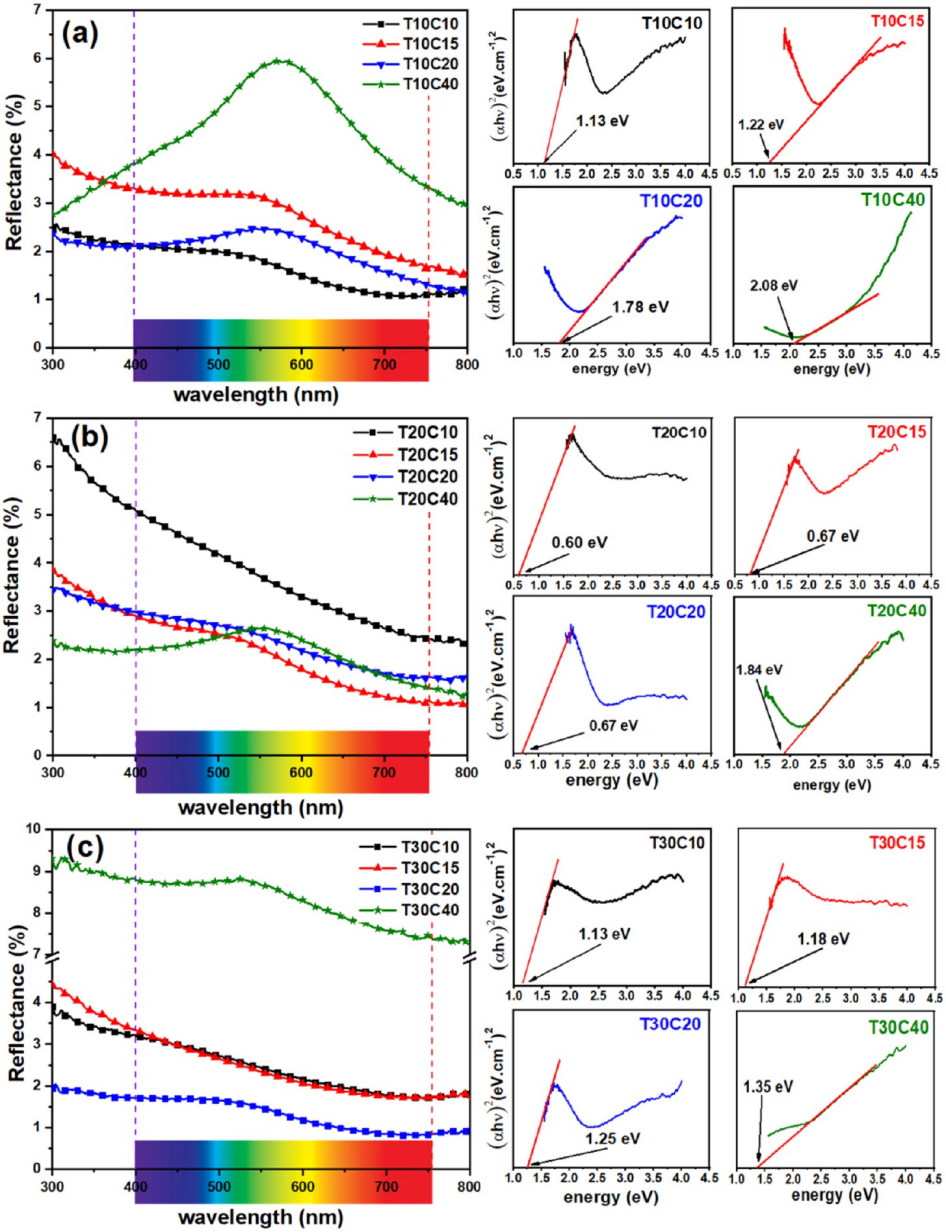
Diffuse reflectance and bandgap of the synthesized samples with (a) 0.1 M, (b) 0.2 M, and (c) 0.3 M TU.

In the visible range (400–751 nm), located on the abscissa of the reflectance graphs, it can be seen that the particles absorb more radiation (less reflectance) in the NIR red range, resulting in low band gap (BG) values, except sample T10C40, which shows different behavior. The BG values are between 0.60 and 2.08 eV, which agrees with the values reported in the literature.^25^ The lower band gap values indicate that the material can be excited by less energetic radiation, generating electron–hole pairs (e^–^/h^+^), which are important for photocatalytic activity.^51^ However, structural defects such as vacancies and substitution of sulfur sites for oxygen can produce intermediate band gaps, which act as traps for charge carriers, resulting in undesirable recombinations.^15^

Samples T10C10 and T30C20 showed the lowest reflectance values across the visible spectrum, indicating a more radiation-absorbing behavior with band gaps of 1.13 and 1.25 eV, respectively. In addition, the brochantite content present in the sample contributes to the increase in the band gap. The increase in the percentage of brochantite reported in the X-ray diffraction results for the samples synthesized with 0.10 M thiourea and varying concentrations of CuSO_4_·5H_2_O (Figure 9a) gradually increased from 1.13 to 2.08 eV.

The Covellite samples (T20C10, T20C15, and T20C20) synthesized with 0.20 M TU showed very low bandgap values (between 0.60 and 0.67 eV). This may be due to intermediate band gaps. In this band gap range, the NPs absorb in the NIR radiation range, contributing to the photothermal effect and consequent optimization of photocatalytic activity.^52^

The band gap values presented in Figure 11 show an increasing behavior of the interval between the valence band (VB) and the conduction band (CB). This means that this optical-electrical property can be adjusted according to the concentrations of the reagents used in the particle synthesis process.

Figure 12 shows the results of the extinction coefficient *k* and refractive index η of the powders produced containing Covellite in their composition. These properties are directly associated with the material’s absorption capacity and the radiation’s interaction time with the sample due to the change in the speed of light as it passes through the material, respectively. Thus, the higher *k* values (Figure 12a) in the NIR region indicate a greater absorption of light, which decreases with the increase in energy (decrease in λ) of the incident photons. This reduction in absorption corresponds to the loss of light due to effects such as scattering.^37^ All the samples shown in Figure 9a showed very similar behavior. However, T30C20 showed slightly higher k values, meaning greater light absorption.

**Figure 12:**
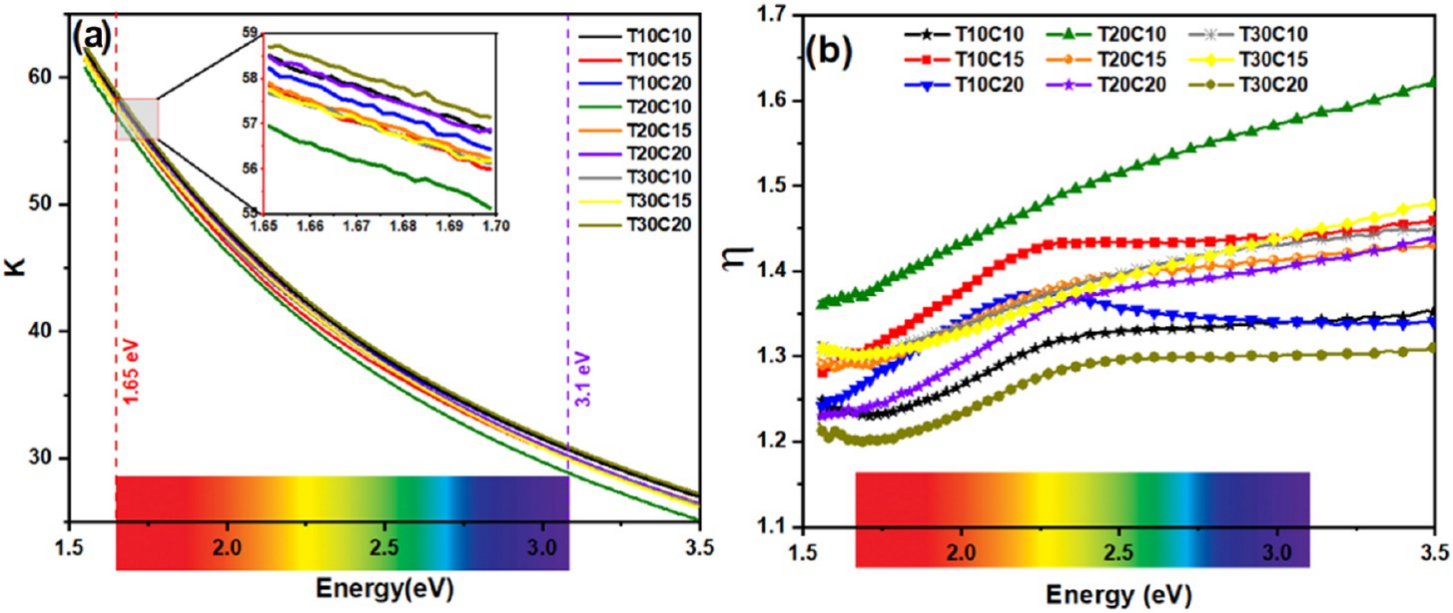
Extinction coefficient *k* (a) and refractive index η (b) of the samples with Covellite.

The behavior of the refractive index in relation to the wavelength shown in Figure 12b complements the optical analysis made previously because although there is greater absorption in the NIR region, the reduction in η means that the speed of light in this wavelength range increases, making the light-NPs interaction faster (η = *c*/*v*). Therefore, the results show that the T20C10 sample has a higher η in this region and, therefore, has a longer light-NP interaction, enabling a more efficient photocatalytic effect.^22^ Therefore, this set of optical results confirms the capacity for photocatalytic activity of nanoparticles composed of Covellite. However, structural defects inherent to the production process are important in promoting high performance.

To aid understanding of the electronic structure and defects in the semiconductor, as well as the recombination of charge carriers (electron–hole), photoluminescence (PL) spectroscopy was analyzed.^53^ Figure 13 shows emission peaks in the visible region of blue (443, 467, 480, and 492 nm) and green (515 and 534 nm). According to Chaki et al. (2014), blue emission is associated with a direct transition between the valence band and the conduction band, characterizing a band–band recombination process. Green emission, on the other hand, results from recombination between electrons located in the conduction band and holes associated with acceptor states, which are linked to copper vacancies in the material.^54^ Therefore, the intensity of the PL spectrum peaks is related to the rapid recombination of electron–hole pairs, thus being an important indicator of the photocatalytic activity capacity of the material.^55^ However, this is not absolute, as other parameters previously presented influence the chemical and electrical interaction of the particles with the dye solution. According to Rafizadeh et al. 2024, the intensity and nature of the emission spectra are also affected by the morphology, size, and dimensional variations of the particles.^12^ Furthermore, it is reported that CuS particles exhibit plasmonic and photothermal properties, enabling them to absorb light energy and convert it into heat efficiently. This means that part of the radiation absorbed by the particles during PL spectroscopy does not result in the transfer of electrons from the excited state to the ground state, emitting light.^56,57^

**Figure 13:**
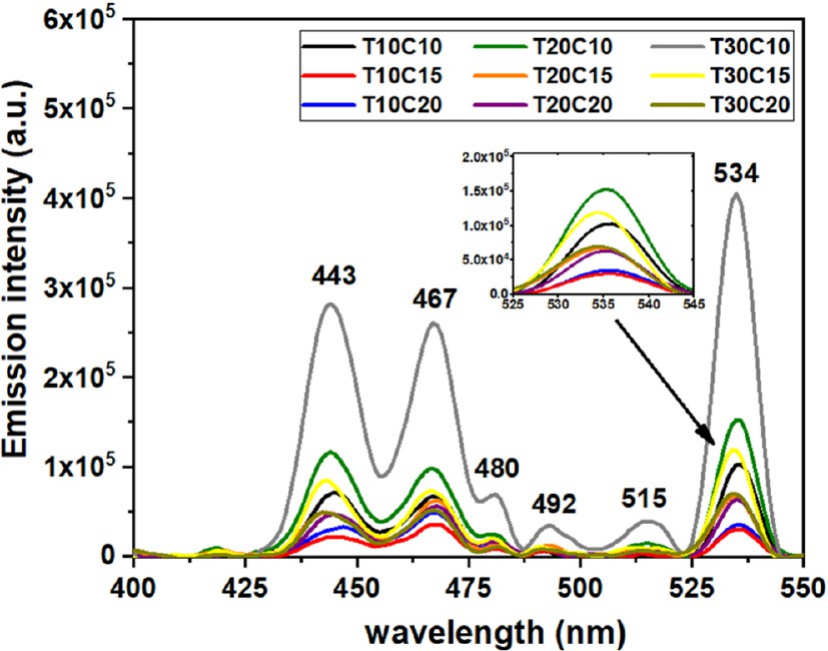
PL emission spectra with a λ_Exc_ = 325 nm for the synthesized samples.

Sample T30C15 shows emission peaks with higher intensities, indicating faster pair recombination than the other samples. However, this same sample has the largest specific surface area (39.6 nm) and pore size of approximately 8.0 nm (see Figure 10a and c). This means that some parameters favor while others may impair the degradation capacity of particles when subjected to visible light. Therefore, further research on the influence of these parameters on the photocatalytic activity of CuS particles still needs to be conducted.

The potential photocatalytic activity of the nanoparticles was investigated through the degradation of the contaminant methylene blue under specific conditions. Low-power visible light (10W), a ratio of 20 mg/100 mL (CuS NPs/MB solution), mechanical stirring, and the nonuse of hydrogen peroxide (H_2_O_2_) enabled a more focused analysis of the NPs’ adsorption and catalysis activity, facilitating easier applicability.

Figure 14 shows the degradation results of the samples, except those synthesized with 0.40 M copper sulfate, due to the standard presence of Brochantite. The methylene blue solution exhibits a broad absorption band spanning wavelengths from 525 to 725 nm, with a maximum peak at 667 nm. The reduction of this peak recorded by UV–vis absorption measurement allows an analysis of MB degradation. Thus, according to Figure 14a, it can be seen that the NPs with a concentration of 0.10 M copper sulfate (T10C10, T20C10, and T30C10) showed a more significant reduction in concentration when illuminated, indicating that when irradiated by visible light, even at low power, the electrons photogenerated from the CuS nanoparticles can be transferred from the valence band to the conduction band, allowing the formation of “holes.” According to Hu et al., 2018, adsorption also contributes to photocatalysis because the adsorbed ^•^O_2_ radical can capture electrons to produce −O_2_^–^ radicals. On the other hand, the holes react with OH^–^ to form the highly oxidizing hydroxyl radicals (−OH) responsible for the degradation of the MB dye.^58,59^ OH^–^ radicals can be generated by the reaction between the H_2_O molecules and the photogenerated holes in the valence layer (h^+^). This effect is usually enhanced or even dependent on the use of hydrogen peroxide (H_2_O_2_), which inhibits the recombination of electron–hole pairs.^19^ However, the absence of H_2_O_2_ showed that the internal electric field produced in the semiconductor by the radiation resulted in a lifetime of charge carriers sufficient to promote degradation reactions of the MB solution.

**Figure 14:**
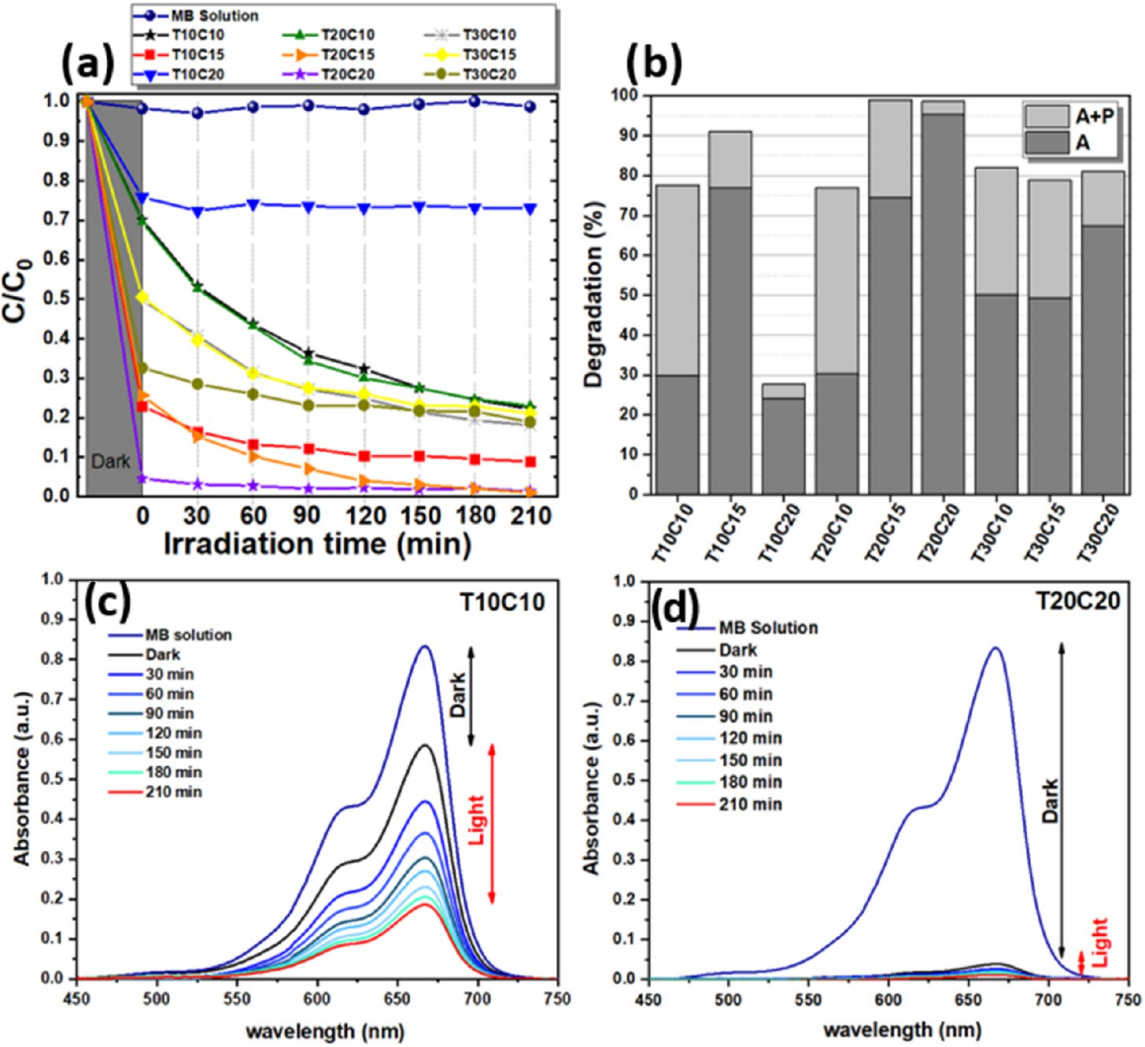
Results of the degradation of MB solution caused by CuS NPs: (a) Concentration, (b) Percentage of adsorption (A), photodegradation (P), the absorbance of samples, (c) T10C10, and (d) T20C20.

Except for sample T30C15, which behaved similarly to those synthesized with 0.40 M copper sulfate, the other samples showed a less intense reduction in *C*/*C*_0_. For example, samples T10C20 and T20C20 exhibited approximately constant behavior up to 210 min of analysis, indicating that higher concentrations of copper sulfate impede the photocatalytic effect of the NPs produced.

Figure 14b shows the percentage contribution of adsorption during the first 30 min in which the MB solution interacted with the NPs in the dark and the subsequent combination of the remaining adsorption effect and the photocatalytic activity of the NPs when subjected to visible light. The percentage of methylene blue adsorbed depends, among various factors, on the size of the NPs, as mentioned above. Small NPs result in larger contact surface areas and, consequently, better adsorption of MB. However, the plasmonic effects resulting from these nanometric dimensions and the negative charge density on the surface of the semiconductor resulting from the pH of the MB solution also influence this effect, as Gupta et al., 2012.^60^ Sample T20C20 adsorbed 95% of the MB molecules, respectively, showing excessive degradation in the first 30 min of the test. This made the subsequent photocatalysis process less significant, as the high adsorption created a dense layer on the NPs, preventing light penetration and consequently reducing the photocatalysis efficiency.^61^

After the initial adsorption period (A), represented by the dark gray portion of the columns in Figure 14b, the mixture (MB solution + NPs) was illuminated for 210 min under mechanical agitation. In this phase of the degradation test, adsorption and photodegradation of the MB solution occurred simultaneously (A+P). Although part of the NPs was covered in MB molecules, the light was transmitted into the NPs, producing the photocatalysis effect, as seen in Figure 14b (light gray part of the columns). During this phase, adsorbed dye molecules can also be degraded and detached from the surfaces of the NPs, causing light transmittance and photocatalysis to continue.^60^ As a result, samples T20C15 and T20C20 stood out, showing degradation of approximately 99% in 210 min of analysis. Figure 14c and d exemplify the situations of adsorption, and high photocatalytic activity of sample T10C10 and high adsorption of sample T20C20 discussed above.

The pseudo-first-order model was used to investigate the initial photocatalytic reaction kinetics of semiconductor particles in the MB solution degradation process.^62^ The linear equation is expressed as

8 $$\ln\left(\frac{C_{0}}{C}\right)=\mathrm{kt}$$

Where *k* is the rate constant (slope) of the pseudo-first-order model, which, as a result of applying the natural logarithm function, expresses the initial degradation rate of the MB solution due to the photocatalytic effects of the CuS particles. Figure 15 shows that, although samples T10C10 and T20C10 showed a greater reduction in concentration during the photodegradation test (Figure 14a), sample T20C15 exhibited the steepest slope of the line obtained by fitting the pseudo-first-order kinetic model. This result indicates that T20C15 showed a higher degradation rate in the initial moments of irradiation, that is, a more intense initial photocatalytic activity. Despite the high adsorption of this sample, the subsequent photocatalytic activity can be explained by the wide distribution of pore sizes presented in the BJH results (Figure 10). However, it is observed that the degradation curve of sample T20C15 tends to stabilize more quickly compared to samples T10C10 and T20C10, which maintained photodegradation efficiency throughout the entire exposure period.

**Figure 15:**
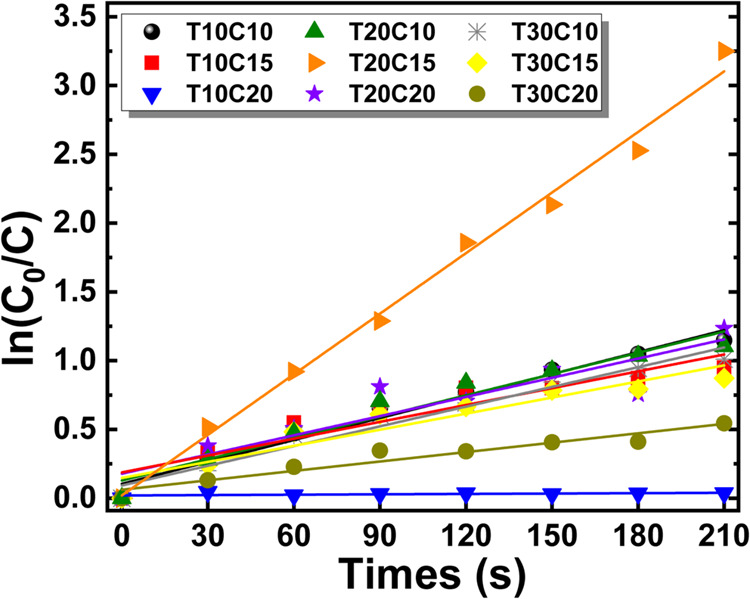
Pseudo-first-order kinetic graph for photocatalytic degradation of CuS particles.

Table 4 shows the values of *k* and the quadratic adjustment *R* ^2^ values for the samples analyzed. The fit is indicative of the model’s adequacy to the observed experimental photocatalysis behavior.^63^ The *R* ^2^ results show that samples T10C10, T20C10, T20C15, and T30C15 exhibited typical catalytic behavior in accordance with the pseudo-first-order model. On the other hand, sample T10C20 diverges from this model. This can be observed in Figure 9a and b, where insignificant degradation is recorded despite the high adsorption presented.

**Table 4:** Values of the Constant *k* and the *R* ^2^ Adjustment of the Pseudo-First-Order Model

samples	*k* (min^–1^)	*R* ^2^
T10C10	0.00532	0.97193
T10C15	0.00408	0.86592
T10C20	9.17747 × 10^–5^	0.10418
T20C10	0.00516	0.94322
T20C15	0.01399	0.99143
T20C20	0.00465	0.80676
T30C10	0.00478	0.95786
T30C15	0.0039	0.88575
T30C20	0.00228	0.91803

Mechanisms of dye decomposition using heterogeneous catalysts have been extensively investigated.^64^ Figure 16 illustrates the photodegradation process. Initially, dye molecules are adsorbed onto the surface of the photocatalyst. Upon radiation exposure, the CuS nanoparticles become photoexcited, promoting electrons from the valence band to the conduction band, thereby generating electron–hole pairs. The promoted electrons migrate to the surface of the catalyst, where they reduce the dissolved oxygen (O_2_^–^). Simultaneously, the photogenerated holes oxidize the water molecules adsorbed on the surface, leading to the formation of hydroxyl radicals (OH−). These radicals, being highly reactive oxidizing agents, attack the dye molecules and supposedly promote their degradation through subsequent chemical reactions:^25^

9 $$C_{16}H_{18}N_{3}{\mathrm{SCl}}_{\mathrm{aq}}\to 16{\mathrm{CO}}_{2}+6H_{2}O+H_{2}{\mathrm{SO}}_{4}+3{\mathrm{HNO}}_{3}+\mathrm{HCl}$$

**Figure 16:**
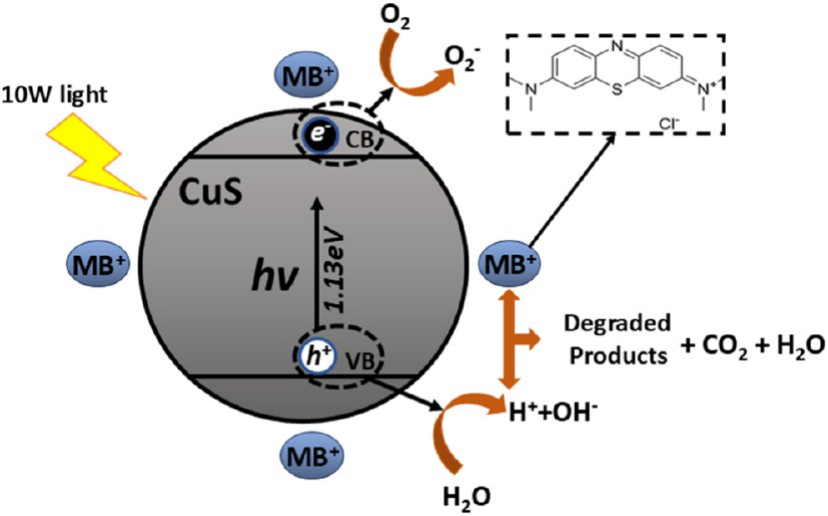
Schematic representation of dye adsorption, photoinduced generation of electron–hole pairs, and the photodegradation process of methylene blue (MB) on the CuS surface.

## Conclusion

The chemical bath synthesis using thiourea and copper sulfate pentahydrate led to the formation of CuS (covellite) and Cu_4_(OH)_6_SO_4_ (brochantite), with structural and morphological variations depending on precursor concentrations. XRD, Raman spectroscopy, SEM-EDS, and STEM analyses confirmed the formation of nanometric covellite (4.1–6.2 nm) and brochantite crystals, especially in samples with higher copper sulfate content, while lower concentrations favored purer covellite, enhancing optical-electronic performance.

BET results showed specific surface areas ranging from 4.99 to 39.6 m^2^/g, with the highest values being characteristic of materials with a high surface area-to-volume ratio, as typically observed in nanoparticles. Additionally, nanoparticle agglomerates led to the formation of meso- and macropores, which contributed to the adsorption of methylene blue. Diffuse reflectance analysis indicated increased absorption in the Vis-NIR region, with bandgap values ranging from 1.13 to 1.78 eV for samples with 0.1 to 0.3 M thiourea, favoring photocatalysis under visible light, and from 0.60 to 0.67 eV for samples with 0.2 M thiourea, supporting photothermal activity. PL spectra revealed partial electron–hole recombination, particularly at 534 nm, associated with copper vacancies.

Photodegradation tests confirmed that varying reagent concentrations influenced particle performance in both adsorption and photocatalysis of methylene blue molecules under low-power visible light. Samples T10C10 (48%) and T20C10 (46%) showed high photocatalytic activity even under MB saturation conditions and without the use of H_2_O_2_ to inhibit charge carrier recombination. In contrast, sample T20C20 exhibited 95% methylene blue adsorption within just 30 min of contact with the solution.

Additionally, the pseudo-first-order kinetic analysis showed that sample T20C15 exhibited the highest degradation rate constant (*k* = 0.01399 min^–1^), with excellent linear correlation (*R* ^2^ = 0.99143), indicating a more intense initial photocatalytic response. In contrast, samples T10C10 and T20C10, although presenting slightly lower rate constants (*k* = 0.00532 and 0.00516 min^–1^, respectively), maintained more stable and prolonged photocatalytic activity, as evidenced by their high *R* ^2^ values (0.97193 and 0.94322).

These results demonstrate that the adopted synthesis process is capable of producing efficient and economically viable CuS nanoparticles for the degradation of industrial organic pollutants, even under low visible light intensity, without additional reagents, and with low catalyst concentration per liquid volume.
